# Neurophysiological Correlates of Central Fatigue in Healthy Subjects and Multiple Sclerosis Patients before and after Treatment with Amantadine

**DOI:** 10.1155/2015/616242

**Published:** 2015-07-05

**Authors:** Emiliano Santarnecchi, Simone Rossi, Sabina Bartalini, Massimo Cincotta, Fabio Giovannelli, Elisa Tatti, Monica Ulivelli

**Affiliations:** ^1^Department of Medicine, Surgery and Neuroscience, Neurology and Clinical Neurophysiology Section, Brain Investigation & Neuromodulation Lab. (Si-BIN Lab), University of Siena, 53100 Siena, Italy; ^2^Berenson-Allen Center for Noninvasive Brain Stimulation, Beth Israel Medical Center, Harvard Medical School, Boston, MA 02215, USA; ^3^Unit of Neurology, Azienda Sanitaria di Firenze, 50121 Florence, Italy

## Abstract

In ten healthy subjects and in ten patients suffering from Multiple Sclerosis (MS), we investigated the cortical functional changes induced by a standard fatiguing repetitive tapping task. The Cortical Silent Period (CSP), an intracortical, mainly GABA_B_-mediated inhibitory phenomenon, was recorded by two different hand muscles, one acting as prime mover of the fatiguing index-thumb tapping task (First Dorsal Interosseous, FDI) and the other one not involved in the task but sharing largely overlapping central, spinal, and peripheral innervation (Abductor Digiti Minimi, ADM). At baseline, the CSP was shorter in patients than in controls. As fatigue developed, CSP changes involved both the “fatigued” FDI and the “unfatigued” ADM muscles, suggesting a cortical spread of central fatigue mechanisms. Chronic therapy with amantadine annulled differences in CSP duration between controls and patients, possibly through restoration of more physiological levels of intracortical inhibition in the motor cortex. These inhibitory changes correlated with the improvement of fatigue scales. The CSP may represent a suitable marker of neurophysiological mechanisms accounting for central fatigue generation either in controls or in MS patients, involving corticospinal neural pools supplying not only the fatigued muscle but also adjacent muscles sharing an overlapping cortical representation.

## 1. Introduction

Fatigue is intrinsic to Multiple Sclerosis (MS) and represents the most common symptom experienced by patients along the course of the disease [1], contributing to disability and to the worsening of their daily quality of life [2, 3]. It may originate at multiple levels of the nervous system “*beginning with ideation of an activity within the cortex and ending with the process of muscle contraction and force generation*” [4].

Such a biological complexity reflects the several, not mutually exclusive, mechanisms that have been invoked to explain the pathophysiology of fatigue in MS, altogether pointing to its central origin: (i) dysfunctions of inhibitory intracortical mechanisms [5, 6], (ii) increased corticomotor excitability [7], and (iii) impaired drive to the primary motor cortex (M1) [8], likely due to (iv) failure of motor programming upstream to pyramidal tracts [9], possibly because of (v) a less synchronous recruitment of corticospinal neurons [10]. Atrophy of cortical layers and white matter [11], reduced perfusion of grey nuclei at the subcortical level [12], diffuse axonal loss [13], and alterations of connectivity [14] may also contribute to generation of central fatigue.

Most of neurophysiological studies on fatigue used Transcranial Magnetic Stimulation (TMS) of the M1, since the amplitude of the motor evoked potential (MEP) directly reflects the excitatory corticospinal output drive [15, 16]. In these studies, results have been variable, since fatiguing exercises reduced [6, 17–19], left unaltered [8, 20], or increased MEP size [21] in MS patients with fatigue. However, changes of the MEP size following fatiguing tasks do not necessarily reflect exclusive cortical activity, but they also include excitability changes occurring in the proximal axonal segment of the spinal motoneuron [22]. Besides giving origin to the excitatory MEP, single-pulse TMS of the M1 elicits also an inhibitory response in the contralateral hand muscles. This phenomenon, called Cortical Silent Period (CSP), refers to the transient interruption of the ongoing voluntary electromyografic (EMG) activity following the excitatory response [23–25]. Spinal mechanisms contribute to the early stage of EMG suppression, while the second part is thought to be caused by a suppression of corticospinal output at the M1 level (for review, see [26]). However, CSP duration is modulated by various cortical and subcortical areas that project to the M1 [27, 28], with converging evidence also indicating how CSP mostly reflects the activity of GABA_B_-ergic circuits [29, 30].

Due to the likely relevance of dysfunctions of intracortical inhibitory mechanisms in the generation of central fatigue [5, 6], we reasoned that the CSP could represent a suitable neurophysiological variable to address whether a fatiguing task involving a hand muscle modifies the inhibitory corticospinal drive. Therefore, the aims of the current study can be summarized as follows. (1) The first aim of the study is to verify CSP changes in healthy controls and MS patients before and after a fatiguing task (repetitive index-thumb tapping); crucially, CSP was recorded by two hand muscles, one acting as prime mover of the tapping task and the other one being not involved in the task while sharing largely overlapping central, spinal, and peripheral innervation. (2) The second aim of the study is to investigate possible relationships between neurophysiological modifications and the score at subjective fatigue scales (Fatigue Severity Scale) [31] or scales measuring the impact of fatigue on quality of life (Fatigue Impact Scale) [32], also taking into account possible aggravating factors as diurnal sleepiness (Epworth Sleepiness Scale, ESS) [33]. (3) The third aim of the study is to verify the effects of chronic therapy of amantadine, one of the symptomatic therapeutic options for central fatigue [34, 35] in patients, both on CSP changes and on fatigue scales. Indeed, a recent pilot, randomized blind study suggested that amantadine, but not other symptomatic drugs such as Modafinil and acetyl-L-carnitine, significantly improved fatigue symptoms in patients with relapsing-remitting MS [36]. Several, still poorly known, mechanisms may potentially account for the beneficial effects of amantadine on fatigue: they include dopaminergic and glutamatergic actions, antiviral or immunologically mediated effect, and amphetamine-like activity [37]. Neurophysiological studies investigating effects of amantadine on central fatigue in MS are still lacking, although they may represent a valuable tool to elucidate some of these mechanisms.

## 2. Subjects and Methods

### 2.1. Patients and Control Group

Ten right-handed female MS patients (mean age 35.9 years; SD 12.3, range 22–64 years) were finally selected from the clinical database of the Neurology and Clinical Neurophysiology Unit at Le Scotte Medical Center in Siena (Italy) (fifty consecutive patients were screened) and included in the study according to the following strict inclusion/exclusion criteria. Inclusion criteria were diagnosis of clinically defined MS [38], either with a relapsing-remitting (*n* = 8) or progressive (*n* = 2) course of the disease; Expanded Disability Status Scale (EDSS) [39] score ranging from 1 to 5.5 (mean EDSS in our sample = 2.1; SD 1.4), so that patients were fully able to perform the requested fatiguing task with their right hand; normal central conduction time, implying normal myelination and conductivity properties for corticospinal fibers directed to the right hand muscles. Five patients did not take long-term drugs; five patients were under treatment with *β*-interferon from more than three months (Table 1). Exclusion criteria were disease relapse in the last two months; presence of other central or peripheral nervous system diseases; presence of Axis I psychiatric diseases according to DSM-IV-R criteria, including anxiety and depression. The control group included 10 healthy right-handed volunteers (3 males and 7 females), with age range of 24–44 years (mean 32.4; SD 6.7). Both patients and controls fulfilled all safety criteria for TMS investigations [40, 41] and had given their written informed consent for the study, which had been approved by the local Institutional Review Board.

### 2.2. Fatiguing Task

We used a standard, easily and ecologically applicable motor task corresponding to the index-thumb tapping test, as defined in part-III of the Unified Parkinson's Disease Rating Scale (UPDRS) [42]. In this task, the First Dorsal Interosseous (FDI) muscle acts as prime mover in conjunction with the flexors of the elbow, while the Abductor Digiti Minimi (ADM) muscle is not involved in task. However, both muscles share similar cortical representations [43, 44], myelomer, and the same peripheral ulnar nerve supply, so that the ADM appears as a suitable control for eventual spread of fatigue at cortical level.

**Figure 1:**
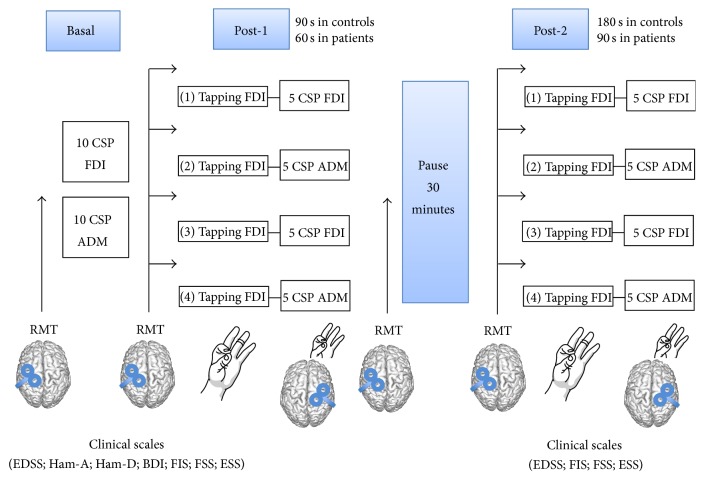
Protocol and time-table of the study. In MS patients, Post-1 and Post-2 blocks were repeated before and after chronic treatment with amantadine 200 mg/die for three months.

Figure 1 depicts the entire protocol. In the same recording session, there were four fatiguing blocks, a pause of about 30 minutes, and additional four fatiguing blocks. Firstly, participants were requested to perform the index-thumb tapping consecutively for 60 seconds (MS patients) or 90 seconds (healthy subjects) (Post-1). At the end of each block, 5 CSP from one of the two muscles were recorded. The same procedure was repeated until 10 CSP from each muscle were obtained. In the second recording session (Post-2), carried out with the same timing of the first one and the same number of CSP per muscle, the tapping task lasted 90 seconds (MS patients) or 180 seconds (healthy controls). The different length of the fatiguing task in patients and controls allowed a comparable level of fatigue at the end of the task for the two groups, since the perceived effort during repeated contractions is higher in patients with MS [19]. Moreover, this prevented the possibility that patients wrongly performed the task at the end of the block due to excessive fatigue. The order of CSP recordings was randomized in both sessions.

### 2.3. Neurophysiological Protocol

Participants sat in a reclining chair, with their forearm resting on the armchairs. TMS of the dominant left motor cortex was produced using a Magstim 200 stimulator (Magstim Co., Whitland, Dyfed, UK) with an 8-shaped coil (external diameter of each loop = 9 cm). The coil handle was kept pointing backward and 45° away from the midline. Once the optimal spot for the right FDI and ADM had been defined, the coil position was marked on the scalp by an inking pen to ensure a correct repositioning during the experiment. The intervals between two consecutive TMS pulses were at least 7 seconds and differed pseudorandomly. Electromyographic signals were recorded through surface electrodes, placed in a belly-tendon montage on the right FDI and ADM muscles, by a four channel EMG system (bandpass 2–1.000 Hz, sampling rate 5 KHz). The ground electrode was positioned on the volar aspect of the forearm.

For CSP studies, analysis was carried out on 500 ms after-stimulus and 100 ms prestimulus windows. Firstly, we determined the resting motor threshold (RMT) according to a standard procedure [15, 40] searching for the minimal intensity eliciting MEPs with 50% probability in 1% increments of the TMS pulse intensity. For the CSP study, stimuli were delivered, while the subject performed a unilateral isometric contraction of the right target muscle (according to the order explained in the previous paragraph) at about 50% of maximum voluntary contraction. The CSP from the FDI and from the ADM was recorded separately (see Figure 1). Stimulus intensity was set at 120% of resting motor threshold (RMT) [45], which was repeatedly checked throughout the session. Since intraindividual CSP changes could merely reflect possible RMT modifications across the recording session, and due to the fact that the CSP duration increases with the stimulus strength [15], besides normalizing the stimulus strength according to possible RMT changes, the CSP was also tested at stimulus intensity of 120% RMT as determined before the fatiguing task.

RMT repeatedly checked throughout the task (see Figure 1) changed neither in controls nor in patients, so these results are not reported. Single trials were rectified and averaged off-line and the resulting trace was used for measurements. The CSP duration was calculated from the negative peak of the MEP to the point when the post-MEP signal reached again 20% of the mean prestimulus EMG level [46]. CSP recordings were carried out in basal conditions and following the two progressively fatiguing tasks based on the index-thumb tapping with the timing described in the previous paragraph (see Figure 1). In the MS patients group, the whole neurophysiological protocol and clinical variables were again sampled after three months of therapy with amantadine 100 mg/die × 2 (see Figure 1).

### 2.4. Evaluation of Central Fatigue and Other Clinical Variables

At the beginning of each recording session before and after the treatment with amantadine, patients were requested to compile subjective scales measuring the severity of fatigue (Fatigue Severity Scale, FSS) [31], the impact of fatigue on the daily quality of life (Fatigue Impact Scale, FIS) [47], and the level of diurnal sleepiness (Epworth Sleepiness Scale, ESS) [33], another variable that could theoretically influence the level of central fatigue. With the aim of evaluating subthreshold depressive and anxiety symptoms, beyond exclusion criteria, we also administered the 14-item Hamilton scale for depression [48] (HAM-D), the 21-item Hamilton scale for anxiety [49] (HAM-A), and the Beck depression inventory [50] (BDI).

### 2.5. Data Analysis

Since the CSP from the FDI and ADM muscles were recorded separately (Figure 1), two separated repeated measures ANOVAs were conducted. A first analysis has been performed to evaluate differences in CSP duration between healthy controls and MS patients before amantadine treatment. Namely, CSP mean durations were entered in mixed ANOVAs with a repeated measures design, with Group (controls versus MS patients) as between-subjects factor and Session (3 levels: Baseline, Post-1, and Post-2) as within-subject factor. Then, in order to test the effect of the amantadine treatment on the CSP in MS patients, the dependent variable (i.e., CSP mean duration) was entered in two-way repeated measures ANOVAs with Session (3 levels: Baseline, Post-1, and Post-2) and Time (before and after the amantadine treatment) as within-subject factors. Finally, a mixed ANOVA has been used to compare MS patients after the amantadine treatment and healthy controls. To correct violations of the sphericity assumption, Greenhouse-Geisser corrections were applied when necessary. Post hoc tests were performed using paired *t*-test and Bonferroni correction for multiple comparisons. The level of significance for all tests was set at *p* < 0.05.

In MS patients, the variation of clinical scale scores before and after amantadine treatment was quantified by the Wilcoxon matched-pairs signed rank test. Correlations between percentage changes of FIS, FSS, and ESS scores with the percentage change of CSP duration in MS patients before and after treatment with amantadine were carried out using Spearman's rho test.

## 3. Results

**Figure 2:**
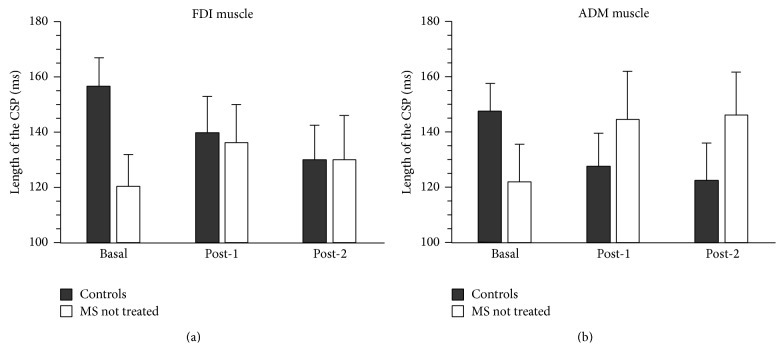
Histograms represent the mean (± SE) duration of the cortical silent period (CSP) from the FDI muscle (a) and ADM muscle (b) in basal conditions and following two progressively fatiguing tasks (Post-1 and Post-2) both in controls and in MS patients before amantadine treatment. Statistics of the repeated measures ANOVA are reported in the text.

**Figure 3:**
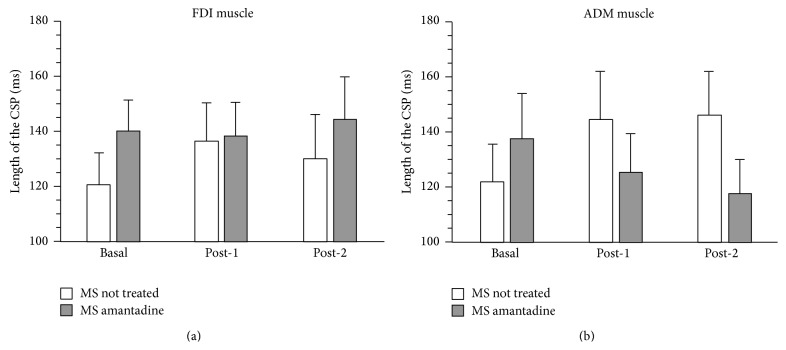
Histograms represent the mean (± SE) duration of the cortical silent period (CSP) from the FDI (a) and ADM (b) muscles at basal conditions and following two progressively fatiguing tasks (Post-1 and Post-2) recorded in MS patients before and after the amantadine treatment. Statistics of the repeated measures ANOVA are reported in the text.

Figures 2 and 3 summarize results, which are herein described point-by-point according to the different questions addressed in the study.

### 3.1. Comparison between Healthy Controls and MS Patients without Treatment

#### 3.1.1. FDI Muscle

No main effects of Group (*F*_1,18_ = 0.558, *p* = 0.465) and Session (*F*_2,36_ = 1.930, *p* = 0.771) were seen, whereas the interaction between these two factors was significant (*F*_2,36_ = 8.719, *p* = 0.002). Post hoc comparisons revealed that the CSP recorded in MS patients before amantadine administration was significantly shorter compared to healthy controls (mean ± SD: 120.3 ± 37.1 ms versus 156.6 ± 33.0 ms; Bonferroni-corrected alpha level: *p* = 0.033), whereas no significant differences emerged after both fatiguing tasks (Figure 2). As for the healthy controls, CSP duration decreased after the fatiguing tasks with respect to the Baseline (mean ± SD: Post-1, 139.8 ± 40.9 and Post-2, 129.9 ± 40.1, Bonferroni-corrected alpha level for Post-1 and Post-2 conditions: *p* = 0.033 and *p* = 0.033, resp.). In contrast, an increase of CSP was observed in MS patients (mean ± SD: Post-1, 136.2 ± 44.1 and Post-2, 130.1 ± 51), although the effect reached significance only in the Post-1 condition (Bonferroni-corrected alpha level: *p* = 0.028) (Figure 2).

#### 3.1.2. ADM Muscle

No main effects of Group (*F*_1,18_ = 0.072, *p* = 0.792) and Session (*F*_2,36_ = 0.050, *p* = 0.906) were seen, whereas the interaction between these two factors was significant (*F*_2,36_ = 11.830, *p* = 0.001). Post hoc comparisons revealed that, similarly to the results observed for the FDI muscle, in healthy controls CSP duration decreased after fatiguing tasks with respect to the Baseline, although the effect was significant only in the Post-2 condition (Bonferroni-corrected alpha level: *p* = 0.038) (Figure 2). In contrast, MS patients showed a significant increase of the CSP compared to the Baseline in the Post-2 condition (Bonferroni-corrected alpha level: *p* = 0.049) with a trend toward significance also for the Post-1 condition (*p* = 0.053) (Figure 2). At Baseline, the mean CSP duration in MS patients was shorter compared to healthy controls (mean ± SD: 121.6 ± 43.0 ms versus 147.5 ± 31.2 ms, resp.) but such difference did not reach statistical significance (*p* = 0.141) (Figure 2).

### 3.2. Effects of Amantadine on the CSP in MS Patients

#### 3.2.1. FDI Muscle

Before the amantadine treatment, the CSP duration was 120.3 ± 37.1 ms, 136.2 ± 44.1 ms, and 130.1 ± 51.0 ms for Baseline, Post-1, and Post-2 conditions, respectively. After the amantadine treatment, the CSP duration was 140.0 ± 36.4 ms, 138.5 ± 37.7 ms, and 144.2 ± 49.2 ms (Figure 3). Repeated measures ANOVA showed that the main effects of Session (*F*_2,18_ = 1.836, *p* = 0.204) and Time (*F*_1,9_ = 0.775, *p* = 0.402) and the interaction between these two factors (*F*_2,36_ = 2.478, *p* = 0.112) were not significant (Figure 3).

#### 3.2.2. ADM Muscle

Before the amantadine treatment, the CSP duration was 121.6 ± 43.0 ms, 144.3 ± 55.0 ms, and 145.9 ± 49.1 ms for Baseline, Post-1, and Post-2 conditions, respectively. After the amantadine treatment, the CSP duration was 137.5 ± 51.9 ms, 124.8 ± 44.9 ms, and 117.2 ± 39.8 ms (Figure 3). The main effects of Session (*F*_2,18_ = 0.373, *p* = 0.694) and Time (*F*_1,9_ = 0.609, *p* = 0.455) were not significant, whereas the interaction between these two factors was significant (*F*_2,36_ = 8.108, *p* = 0.016) (Figure 3). Post hoc comparisons confirmed that before the amantadine treatment CSP duration was shorter for Baseline compared to Post-1 and Post-2 conditions (see results for the “comparison between healthy controls and MS patients without treatment” session), whereas after the amantadine treatment CSP did not differ across conditions. Moreover, no significant differences emerged between CSP durations recorded before and after the amantadine treatment for Baseline, Post-1, and Post-2 conditions.

Mixed ANOVA performed to compare MS patients under treatment with amantadine and healthy controls showed no significant differences in CSP duration for either the FDI and ADM muscles (Bonferroni-corrected alpha level: *p* > 0.050 for all comparisons).

### 3.3. Correlations between Clinical Scales and CSP Changes during Therapy

Fatigue scales improved in most of the patients (Table 1) after chronic therapy with amantadine: the improvement at the FSS was significant (*p* = 0.012), while the significance of the improvement at the FIS was marginal (*p* = 0.059). The ESS did not change significantly. Percentage changes of the CSP duration recorded from the FDI muscle in MS patients before and during chronic treatment with amantadine significantly correlated with improvement at the FIS scale (*p* = 0.039, *r* = 0.673) but not at the FSS and ESS scales. None of the correlations was significant for CSP changes in the ADM muscle.

## 4. Discussion

Results of the current study, the first-ever investigating the neurophysiological effects of amantadine on fatigue in MS patients, will be commented point-by-point in the following dedicated paragraphs.

### 4.1. Comparison between Healthy Controls and MS Patients without Treatment

The fatiguing exercise of a single hand muscle (i.e., index-thumb tapping according to the UPDRS-part III) induces, either in controls or in patients with MS, rapid adapting changes in the inhibitory circuits of the contralateral motor cortex or upstream to it, which spread to other muscles not involved in the fatiguing task (Figure 2). More in detail, the length of the CSP in controls progressively decreased according to the level of fatigue (Post-2 > Post-1), while in drug-free MS patients it increased at Post-1 and at Post-2 in both muscles, despite the fact that the ADM muscle was not engaged in the fatiguing task. At variance with short-interval intracortical inhibition changes, a measure of GABA_A_ activity in the motor cortex [15], which were limited to the “fatigued” FDI muscle and spared the “unfatigued” ADM muscle [51], the presence of consistent CSP changes on both muscles suggests a spread of mechanisms generating fatigue at cortical level, where the neural populations controlling the FDI and ADM muscles are largely overlapping [43, 44]. Such a lack of selectivity of CSP adapting changes to the fatiguing exercise (which is in keeping with the notion that, as fatigue develops, there is a spread of muscle activation beyond the target muscle [51–53]) may plausibly account for the positive correlation of CSP changes with the improvement of subjective fatigue scales in patients whose scores would have been unlikely changed if fatigue would have influenced only one small hand muscle.

In previous studies on healthy subjects [51, 54, 55] or in MS patients with fatigue [19], changes in CSP duration were found to be confined to the fatigued muscle. However, these results are not comparable to the ones presented in this investigation, because CSP was recorded from tibialis anterior [54] or elbow flexors muscles [55] rather than from intrinsic hand muscles that, crucially, are supplied with monosynaptic corticospinal connections. Additionally, we looked at CSP changes occurring* after* the fatiguing task rather than* during* its execution [19, 51] for the following practical reasons: under these circumstances, (i) it is easier to keep a stable level of the isometric contraction preceding the TMS pulse and (ii) it is easier to balance the level of effort between controls and patients.

As fatigue developed (i.e., Post-2 > Post-1), there was an opposite trend of CSP duration in controls and in patients (Figure 2). Since the late part of the CSP would mainly reflect GABA_b_-mediated mechanisms at the cortical level, disinhibition probably occurs at this level with prolonged tapping in healthy subjects. Indeed, both the repetitiveness of motor commands and sensory feedback signals from the contracting muscles are known to transiently reduce intracortical levels of GABAergic inhibition [56]. This could represent an adaptive process of inhibitory mechanisms at M1 and/or in different cortical and subcortical areas projecting to the M1, aiming at adapting corticospinal drive to the fatigued muscle(s) as well as a possible biological marker of central fatigue. Possibly, also the repetitive activation of group III-IV muscle afferents during sustained tapping might have influenced the responsiveness of the motor cortex to the TMS pulse in line with the notion that these afferents may facilitate central fatigue at least for endurance lower limb exercising [57].

Whatever the mechanism(s) generating central fatigue, it can be hypothesized that such a compensatory downregulation of inhibitory cortical mechanisms might be partly lost in MS patients, at least in the current sample which was selected on the basis of normal conductivity in corticospinal pathways towards the performing hand and with central fatigue. Under these circumstances, as fatigue develops, intracortical inhibitory circuits located within or upstream to M1 might be engaged in an opposite manner. Notably, amantadine restored these adapting changes close to the level of those observed in control subjects (Figure 3) (see later in the discussion).

### 4.2. Differences between Patients and Controls “at Rest”

In basal conditions (i.e., before the fatiguing task), amantadine-free MS patients had a significantly shortened CSP in the FDI muscle than controls (Figure 2), which was in agreement with previous studies in which a shorter duration of the CSP was found in MS patients during disease relapse [57, 58]. In the current study, the chronic therapy with amantadine, which significantly improved scores of fatigue scales, produced a nonsignificant lengthening of the CSP that, however, annulled these differences between MS patients and controls. This indirectly suggests that the length of the CSP, and its “responsiveness” to amantadine even before patients' engagement in the fatiguing task, might represent a piece of the neurophysiological mosaic accounting for mechanisms generating motor fatigue at cortical level.

### 4.3. Effects of Amantadine on the CSP in MS Patients

Chronic therapy with amantadine improved central motor fatigue according to subjective measurements at FIS and FSS but not at the ESS. It also produced an increase of the CSP duration in patients when tested either before or after the fatiguing task, so that the significant difference between patients and controls at Baseline was annulled. The normalization of CSP duration induced by amantadine significantly correlated with the subjective perception of fatigue in the FDI muscle. Thus, CSP might be regarded as a suitable marker to neurophysiologically monitor the effects of symptomatic therapy for central fatigue. Amantadine, by its NMDA-antagonist mechanism [59], might have induced an increase in the physiological patterns of neural pools responsible for CSP, possibly rebalancing the dysfunctional GABA/Glutamate levels at cortical level (i.e., M1 or upstream to M1) due to central fatigue. It is intriguing that amantadine was not able to change the duration of the CSP in normal subjects, at least during an acute challenge [60]. However, this could be due to a sort of ceiling effect in presence of a normal GABA/Glutamate balance. According to this hypothesis, a slight lengthening of CSP duration was seen in healthy volunteers after a single dose of dextromethorphan, another NMDA-antagonist [61].

A previous study documented the lack of CSP changes induced by chronic administration of Modafinil, another fatigue-modifying drug with a non-NMDA antagonism mechanism [62], despite improvement of subjective fatigue rating [63]. This, and other overall conflicting results on intracortical inhibitory and excitatory functions in MS patients with fatigue (see [64] for a review), might simply reflect that these results were obtained in patients with or without fatigue, while participants were “at rest” (i.e., not engaged in fatiguing motor tasks). This implies that eventual differences of cortical excitability due to fatigue should reflect a trait rather than a state. Therefore, testing patients “at rest” could have probably underestimated changes of neurophysiological variables induced by intercurring fatigue immediately after the motor task execution.

### 4.4. Limitations of the Study

The relatively small sample size and the lack of a placebo condition and of randomization are all factors limiting the strength of the conclusions. However, patients included in the current study were carefully selected according to very strict criteria. Objective neurophysiological measurements, as well as a persistent beneficial effect at three months, make the occurrence of possible placebo effects less likely. Moreover, half of the patients were under immunomodulating therapy with Interferon. A subanalysis of results did not show differences in CSP duration between treated and nontreated patients. However, larger samples of patients are needed to confirm this finding.

### 4.5. Conclusions

This is the first study addressing the neurophysiological effects of amantadine on central fatigue in MS patients. Results suggest that chronic treatment with amantadine restores more physiological levels of intracortical inhibition in the motor cortex in patients with MS, expressed as both a normalization of CSP in basal condition and a reduction of CSP duration after the fatiguing task, both associated to an improvement of central fatigue scores.

Interestingly, CSP may represent a suitable index of the neurophysiological mechanisms underpinning central fatigue in general, probably involving corticospinal neural pools directed not only towards the fatigued muscle but also towards adjacent muscles sharing an overlapping cortical representation.

## Figures and Tables

**Table 1:** Demographics and clinical characteristics of MS patients. RR, relapsing-remitting; SP, secondary progressive; EDSS, Expanded Disability Status State; INF, Interferon; No, patients without Amantadine; Th., patients treated with Amantadine; Ham-A, Hamilton anxiety; Ham-D, Hamilton depression; FSS, Fatigue Severity Scale; FIS, Fatigue Impact Scale; ESS, Epworth Sleepiness Scale.

Sex, age	SM type	EDSS	Therapy	Dur.	Ham-A		Ham-D		Beck		FSS		FIS		ESS	
					No	Th	No	Th	No	Th	No	Th	No	Th	No	Th
F, 29	RR	1	No	—	1	1	2	2	3	3	3.8	3.3	84	81	8	9
F, 43	RR	1.5	INF*β*-1a (44 × 3)	7	0	0	1	1	1	1	6.4	6.2	153	144	19	14
F, 46	SP	3	No	—	1	1	1	1	3	2	3.6	3	19	11	5	5
F, 37	RR	1	No	—	0	0	1	0	2	0	6.2	4.3	60	34	15	10
F, 64	SP	5.5	INF*β*-1b	15	0	0	0	0	3	1	6.4	6.7	89	76	10	15
F, 28	RR	1.5	INF*β*-1a (44 × 3)	6	0	0	1	1	4	2	5.8	4.2	98	72	6	3
F, 22	RR	1	INF*β*-1a (22 × 3)	24	3	3	2	2	9	8	7	7	96	102	3	3
F, 33	RR	2.5	No	—	1	2	3	3	6	6	5.8	4.2	98	78	9	9
F, 28	RR	2	No	—	3	3	1	1	8	7	6.2	5	130	90	11	9
F, 29	RR	2	INF*β*-1a (44 × 3)	8	4	4	3	3	4	5	5.8	4	110	85	4	2

Mean		2.1			1.3	1.4	1.5	1.4	4.3	3.5	5.7	4.8^*∗*^	93.7	77.3^*∗*^	9.0	7.9
SD		1.4			1.5	1.5	1.0	1.1	2.6	2.8	1.1	1.4	36.5	35.9	5.0	4.6

^*∗*^ *p* < 0.05, Wilcoxon matched-pairs signed rank test.

## References

[1] Krupp L. (2006). Fatigue is intrinsic to multiple sclerosis (MS) and is the most commonly reported symptom of the disease. *Multiple Sclerosis*.

[2] Bakshi R., Shaikh Z. A., Miletich R. S. (2000). Fatigue in multiple sclerosis and its relationship to depression and neurologic disability. *Multiple Sclerosis*.

[3] Comi G., Leocani L., Rossi P., Colombo B. (2001). Physiopathology and treatment of fatigue in multiple sclerosis. *Journal of Neurology*.

[4] Vucic S., Burke D., Kiernan M. C. (2010). Fatigue in multiple sclerosis: mechanisms and management. *Clinical Neurophysiology*.

[5] Leocani L., Colombo B., Magnani G. (2001). Fatigue in multiple sclerosis is associated with abnormal cortical activation to voluntary movement—EEG evidence. *NeuroImage*.

[6] Liepert J., Mingers D., Heesen C., Bäumer T., Weiller C. (2005). Motor cortex excitability and fatigue in multiple sclerosis: a transcranial magnetic stimulation study. *Multiple Sclerosis*.

[7] Thickbroom G. W., Sacco P., Faulkner D. L., Kermode A. G., Mastaglia F. L. (2008). Enhanced corticomotor excitability with dynamic fatiguing exercise of the lower limb in multiple sclerosis. *Journal of Neurology*.

[8] Sheean G. L., Murray N. M. F., Rothwell J. C., Miller D. H., Thompson A. J. (1997). An electrophysiological study of the mechanism of fatigue in multiple sclerosis. *Brain*.

[9] Morgante F., Dattola V., Crupi D. (2011). Is central fatigue in multiple sclerosis a disorder of movement preparation?. *Journal of Neurology*.

[10] Caliandro P., Padua L., Rossi A. (2014). Jitter of corticospinal neurons during repetitive transcranial magnetic stimulation. Method and possible clinical implications. *Brain Stimulation*.

[11] Riccitelli G., Rocca M. A., Forn C., Colombo B., Comi G., Filippi M. (2011). Voxelwise assessment of the regional distribution of damage in the brains of patients with multiple sclerosis and fatigue. *American Journal of Neuroradiology*.

[12] Inglese M., Park S.-J., Johnson G. (2007). Deep gray matter perfusion in multiple sclerosis: dynamic susceptibility contrast perfusion magnetic resonance imaging at 3 T. *Archives of Neurology*.

[13] De Stefano N., Narayanan S., Francis G. S. (2001). Evidence of axonal damage in the early stages of multiple sclerosis and its relevance to disability. *Archives of Neurology*.

[14] Tartaglia M. C., Narayanan S., Francis S. J. (2004). The relationship between diffuse axonal damage and fatigue in multiple sclerosis. *Archives of Neurology*.

[15] Groppa S., Oliviero A., Eisen A. (2012). A practical guide to diagnostic transcranial magnetic stimulation: report of an IFCN committee. *Clinical Neurophysiology*.

[16] Rossini P. M., Rossi S. (2007). Transcranial magnetic stimulation: diagnostic, therapeutic, and research potential. *Neurology*.

[17] Perretti A., Balbi P., Orefice G. (2004). Post-exercise facilitation and depression of motor evoked potentials to transcranial magnetic stimulation: a study in multiple sclerosis. *Clinical Neurophysiology*.

[18] Petajan J. H., White A. T. (2000). Motor-evoked potentials in response to fatiguing grip exercise in multiple sclerosis patients. *Clinical Neurophysiology*.

[19] Thickbroom G. W., Sacco P., Kermode A. G. (2006). Central motor drive and perception of effort during fatigue in multiple sclerosis. *Journal of Neurology*.

[20] Schubert M., Wohlfarth K., Rollnik J. D., Dengler R. (1998). Walking and fatigue in multiple sclerosis: the role of the corticospinal system. *Muscle Nerve*.

[21] Nielsen J. F., Norgaard P. (2002). Increased post-exercise facilitation of motor evoked potentials in multiple sclerosis. *Clinical Neurophysiology*.

[22] Rossi A., Rossi S., Ginanneschi F. (2012). Activity-dependent changes in intrinsic excitability of human spinal motoneurones produced by natural activity. *Journal of Neurophysiology*.

[23] Cantello R., Gianelli M., Civardi C., Mutani R. (1992). Magnetic brain stimulation: the silent period after the motor evoked potential. *Neurology*.

[24] Fuhr P., Agostino R., Hallett M. (1991). Spinal motor neuron excitability during the silent period after cortical stimulation. *Electroencephalography and Clinical Neurophysiology*.

[25] Inghilleri M., Berardelli A., Cruccu G., Manfredi M. (1993). Silent period evoked by transcranial stimulation of the human cortex and cervicomedullary junction. *The Journal of Physiology*.

[26] Cincotta M., Quartarone A., Abbruzzese G., Miniussi C., Paulus W., Rossini P. M. (2012). Motor cortical and corticospinal measures in health and disease. *Transcranial Brain Stimulation*.

[27] Cincotta M., Borgheresi A., Guidi L. (2000). Remote effects of cortical dysgenesis on the primary motor cortex: evidence from the silent period following transcranial magnetic stimulation. *Clinical Neurophysiology*.

[28] Classen J., Schnitzler A., Binkofski F. (1997). The motor syndrome associated with exaggerated inhibition within the primary motor cortex of patients with hemiparetic. *Brain*.

[29] Stetkarova I., Kofler M. (2013). Differential effect of baclofen on cortical and spinal inhibitory circuits. *Clinical Neurophysiology*.

[30] Siebner H. R., Dressnandt J., Auer C., Conrad B. (1998). Continuous intrathecal baclofen infusions induced a marked increase of the transcranially evoked silent period in a patient with generalized dystonia. *Muscle and Nerve*.

[31] Krupp L. B., LaRocca N. G., Muir-Nash J., Steinberg A. D. (1989). The fatigue severity scale. Application to patients with multiple sclerosis and systemic lupus erythematosus. *Archives of Neurology*.

[32] Fisk J. D., Doble S. E. (2002). Construction and validation of a fatigue impact scale for daily administration (D-FIS). *Quality of Life Research*.

[33] Johns M. W. (1991). A new method for measuring daytime sleepiness: the Epworth sleepiness scale. *Sleep*.

[34] Krupp L. B., Coyle P. K., Doscher C. (1995). Fatigue therapy in multiple sclerosis: results of a double-blind, randomized, parallel trial of amantadine, pemoline, and placebo. *Neurology*.

[35] Ashtari F., Fatehi F., Shaygannejad V., Chitsaz A. (2009). Does amantadine have favourable effects on fatigue in Persian patients suffering from multiple sclerosis?. *Neurologia i Neurochirurgia Polska*.

[36] Ledinek A. H., Sajko M. C., Rot U. (2013). Evaluating the effects of amantadin, modafinil and acetyl-L-carnitine on fatigue in multiple sclerosis—result of a pilot randomized, blind study. *Clinical Neurology and Neurosurgery*.

[37] Pucci E., Branãs P., D'Amico R., Giuliani G., Solari A., Taus C. (2007). Amantadine for fatigue in multiple sclerosis. *Cochrane Database of Systematic Reviews*.

[38] Polman C. H., Wolinsky J. S., Reingold S. C. (2005). Multiple sclerosis diagnostic criteria: three years later. *Multiple Sclerosis*.

[39] Kurtzke J. F. (1983). Rating neurologic impairment in multiple sclerosis: an expanded disability status scale (EDSS). *Neurology*.

[40] Rossi S., Hallett M., Rossini P. M. (2009). Safety, ethical considerations, and application guidelines for the use of transcranial magnetic stimulation in clinical practice and research. *Clinical Neurophysiology*.

[41] Rossi S., Hallett M., Rossini P. M., Pascual-Leone A. (2011). Screening questionnaire before TMS: an update. *Clinical Neurophysiology*.

[42] Fahn S., Elton R., Fahn S., Marsden C. D., Calne D. B., Goldstein M. (1987). Members of the updrs development committee. *Recent Developments in Parkinson's Disease*.

[43] Rossi S., Pasqualetti P., Tecchio F., Sabato A., Rossini P. M. (1998). Modulation of corticospinal output to human hand muscles following deprivation of sensory feedback. *NeuroImage*.

[44] Rossini P. M., Rossi S., Tecchio F., Pasqualetti P., Finazzi-Agrò A., Sabato A. (1996). Focal brain stimulation in healthy humans: motor maps changes following partial hand sensory deprivation. *Neuroscience Letters*.

[45] Rossini P., Burke D., Chen R. (2015). Non-invasive electrical and magnetic stimulation of the brain, spinal cord, roots and peripheral nerves: Basic principles and procedures for routine clinical and research application. An updated report from an I.F.C.N. Committee. *Clinical Neurophysiology*.

[46] Cincotta M., Borgheresi A., Boffi P. (2002). Bilateral motor cortex output with intended unimanual contraction in congenital mirror movements. *Neurology*.

[47] Fisk J. D., Doble S. E. (2002). Construction and validation of a fatigue impact scale for daily administration (D-FIS). *Quality of Life Research*.

[48] Hamilton M. (1967). Development of a rating scale for primary depressive illness. *The British Journal of Social and Clinical Psychology*.

[49] Hamilton M. (1959). The assessment of anxiety states by rating. *The British Journal of Medical Psychology*.

[50] Beck A. T., Steer R. A., Ball R., Ranieri W. F. (1996). Comparison of Beck depression inventories-IA and -II in psychiatric outpatients. *Journal of Personality Assessment*.

[51] Benwell N. M., Mastaglia F. L., Thickbroom G. W. (2007). Differential changes in long-interval intracortical inhibition and silent period duration during fatiguing hand exercise. *Experimental Brain Research*.

[52] Hagbarth K.-E., Bongiovanni L. G., Nordin M. (1995). Reduced servo-control of fatigued human finger extensor and flexor muscles. *The Journal of Physiology*.

[53] Psek J. A., Cafarelli E. (1993). Behavior of coactive muscles during fatigue. *Journal of Applied Physiology*.

[54] McKay W. B., Stokic D. S., Sherwood A. M., Vrbova G., Dimitrijevic M. R. (1996). Effect of fatiguing maximal voluntary contraction on excitatory and inhibitory responses elicited by transcranial magnetic motor cortex stimulation. *Muscle and Nerve*.

[55] Taylor J. L., Butler J. E., Gandevia S. C. (2000). Changes in muscle afferents, motoneurons and motor drive during muscle fatigue. *European Journal of Applied Physiology*.

[56] Ljubisavljević M., Milanović S., Radovanović S., Vukčević I., Kostić V., Anastasijević R. (1996). Central changes in muscle fatigue during sustained submaximal isometric voluntary contraction as revealed by transcranial magnetic stimulation. *Electroencephalography and Clinical Neurophysiology/Electromyography and Motor Control*.

[57] Amann M. (2012). Significance of Group III and IV muscle afferents for the endurance exercising human. *Clinical and Experimental Pharmacology and Physiology*.

[58] Fierro B., Salemi G., Brighina F. (2002). A transcranial magnetic stimulation study evaluating methylprednisolone treatment in multiple sclerosis. *Acta Neurologica Scandinavica*.

[59] Caramia M. D., Palmieri M. G., Desiato M. T. (2004). Brain excitability changes in the relapsing and remitting phases of multiple sclerosis: a study with transcranial magnetic stimulation. *Clinical Neurophysiology*.

[60] Reis J., John D., Heimeroth A. (2006). Modulation of human motor cortex excitability by single doses of amantadine. *Neuropsychopharmacology*.

[61] Ziemann U., Chen R., Cohen L. G., Hallett M. (1998). Dextromethorphan decreases the excitability of the human motor cortex. *Neurology*.

[62] Gerrard P., Malcolm R. (2007). Mechanisms of modafinil: a review of current research. *Neuropsychiatric Disease and Treatment*.

[63] Lange R., Volkmer M., Heesen C., Liepert J. (2009). Modafinil effects in multiple sclerosis patients with fatigue. *Journal of Neurology*.

[64] Yusuf A., Koski L. (2013). A qualitative review of the neurophysiological underpinnings of fatigue in multiple sclerosis. *Journal of the Neurological Sciences*.

